# Tracking the genome of four *Pseudomonas aeruginosa* isolates that have a defective Las quorum-sensing system, but are still virulent

**DOI:** 10.1099/acmi.0.000132

**Published:** 2020-05-19

**Authors:** Enrique Martínez-Carranza, Selene García-Reyes, Abigail González-Valdez, Gloria Soberón-Chávez

**Affiliations:** ^1^​ Departamento de Biología Molecular y Biotecnología, Instituto de Investigaciones Biomédicas, Universidad Nacional Autónoma de México, Ciudad Universitaria, Apdo. Postal 70228, C. P. 04510, CDMX, México

**Keywords:** bacterial evolution, *Pseudomonas aeruginosa*, type three secretion system

## Abstract

In this work we analysed the whole genome extended multilocus sequence typing (wgMLST) of four *Pseudomonas aeruginosa* strains that are characterized by being virulent despite having a defective Las quorum-sensing (QS) system, and compare them with the wgMLST of the PAO1 and PA14 type strains. This comparison was done to determine whether there was a genomic characteristic that was common to the strains with an atypical QS response. The analysed strains include two environmental isolates (ID 4365 isolated from the Indian Ocean, and M66 isolated from the Churince water system in Cuatro Ciénegas Coahuila, México), one veterinary isolate (strain 148 isolated from the stomach of a dolphin) and a clinical strain (INP43 that is a cystic fibrosis pediatric isolate). We determine that the six analysed strains have a core genome of 4689 loci that was used to construct a wgMLST-phylogeny tree. Using the cano-wgMLST_BacCompare software we found that there was no common genomic characteristic to the strains with an atypical QS-response and we identify ten loci that are highly discriminatory of the six strains’ phylogeny so that their MLST can reconstruct the wgMLST-phylogeny tree of these strains. We discuss here the nature of these ten highly discriminatory genes in the context of *P. aeruginosa* virulence and evolution.

## Introduction

*P. aeruginosa* is a wide-spread environmental bacterium, but is also an important opportunistic pathogen. *P. aeruginosa* infections represent a serious health problem due to its high intrinsic and acquired antibiotic resistance [1] and its production of different virulence associated traits [2, 3]. Several of the virulence factors produced by this bacterium, including, elastase (ELA), pyocyanin (PYO) and rhamnolipids (RL), are regulated at the level of transcription by a complex regulatory cascade called quorum-sensing (QS) [4]. The LasR transcriptional regulator constitutes the top of the QS regulatory cascade that when coupled with the autoinducer (AI) 3-oxo-dodecanoyl homoserine lactone (3O-C12-HSL) activates the transcription of ELA and other virulence associated traits. LasR/3O-C12-HSL also activates the transcription of *rhlR* and *rhlI,* which encode a second transcriptional regulator (RhlR) and its cognate AI, butanoyl-homoserine lactone (C4-HSL). In addition LasR/3O-C12-HSL also activates the transcription of the gene encoding PqsR, the transcriptional regulator of the third QS system that uses alkyl quinolones as AI. RhlR/C4-HSL in turn activates the transcription of the genes involved in the production of PYO and RL [4]. It has been reported that *pqsE*, a gene that forms part of the *pqsABCDE* operon that is activated by PqsR, encodes an enzyme that produces an as yet unidentified AI that interacts with RhlR, modifying its transcriptional activity [5]. The virulence of *P. aeruginosa* is not only determined by the QS cascade, but other factors such as the type III secretion system are also involved [6, 7].

The genomic constitution of *P. aeruginosa* is unique since contrary to other bacteria, the virulence associated genes form part of its core-genome [8] and clinical and environmental isolates constitute a single population with high genome conservation [9]. *P. aeruginosa* species contains three clades, two of which are highly similar and the third clade is genetically more diverse [10]. Clade 1 type strain is PAO1, Clade 2 type strain is PA14 and PA7 outlier strain is the type strain of Clade 3. Strains belonging to all clades are geographically diverse and include both clinical and environmental isolates. The only case of endemicity that has been reported are the two clonal groups belonging to Clade 2, which were isolated in 2011 (clonal group 2A) and in 2015 (clonal group 2B) from the Churince water system in Cuatro Ciénegas Coahuila México [11]. The distribution of these two clonal-groups is particular to the Churince system, suggesting that *P. aeruginosa* isolates have been part of the bacterial community of this water system since ancient times.

It has been reported that strains defective in the LasR QS-system are very frequently isolated from patients with *P. aeruginosa* infections [12] and that in many cases these defective strains are able to express QS-dependent virulence factors such as PYO and RL since *rhlR* expression in these strains is independent of LasR [13–15]. Furthermore, it has been proposed that LasR deficient RhlR proficient strains are selected during the time course of an infection under specific conditions [15]. If the *P. aeruginosa* strains presenting an atypical QS response, such as those studied in this work (Table 1), are the product of a determined selective pressure as has been proposed [15], it is plausible that independent isolates sharing this condition will have common genomic characteristics that can be distinguished.

**Table 1.** Characteristics of the four strains with an atypical QS response studied in this work

Strain	Characteristic	LasR deffect	Ref.	RL production	PYO production	Virulence in *Galleria mellonella* model*	Virulence in mouse model†	Ref.
INP43	Cystic Fibrosis pediatric isolate	Contains a *lasR n*on-sense mutation Q45·	This work	+	++	nd	+/-	[17] This work
148	Dolphin stomach isolate	Contains a 20 kb deletion that includes *lasR,* and an IS insertion in *vfr*	[13]	+	+	nd	+	[9]
ID4365	Indian Ocean isolate	Contains a *lasR n*on-sense mutation Q24·	This work	+	++	nd	+	[9]
M66	Churince water-system isolate	Contains an inactive LasR, probably due to the A80V *cyaB* mutation	This work	+	++	+	nd	[11]

*nd, means not done.

†+/- means that when five mice were injected with 10^8^ colony forming units (c.f.u.) it took 48 h for INP43 to kill 4 of 5 mice injected, while the same number of PA14, or PAO1 cells, killed all 5 mice at 24 h of injection.

*P. aeruginosa* strains with atypical QS systems such as the LasR-defective RhlR-proficient strains studied here (Table 1) represent a challenge to the development of anti-virulence therapeutic strategies to treat *P. aeruginosa* infections that is a field of intensive research to search for alternatives to the use of antibiotics [16].

Here we study the genome of four independent *P. aeruginosa* isolates that are defective in the LasR QS-system, but are able to produce PYO and RL and are virulent (Table 1), to determine whether they share some genetic traits that could be used as markers, and compared them with the PAO1 and PA14 type strains. Two of the studied strains are environmental, ID4365 isolated from the Indian Ocean [9] and M66 isolated from the Churince water system in 2011 (belongs to clonal group 2A) [11], one strain was isolated from the stomach of a dolphin [9, 13], and the fourth strain, INP43, is a pediatric Cystic Fibrosis isolate [17]. We focus on the genomic analysis of only these four strains because they have been characterized in terms of their defective Las QS system and their virulence (Table 1), and because they represent independent isolates that include environmental, veterinary and clinical strains. We reasoned that if the genomic analysis of these four *P. aeruginosa* strains with an atypical QS response (Table 1) showed common features, we might be able to identify a common selective pressure and molecular response involved in the generation of LasR defective and RhlR proficient strains. However, no particular genomic profile of these four strains was identified.

To make the genomic comparisons of the four LasR-deficient strains we used the cano-wgMLST_BacCompare software [18] (available at http://baccompare.imst.nsysu.edu.tw), that was reported recently to make epidemiological and comparative genomic analysis of bacteria based on whole genome extended multilocus sequence typing (wgMLST), and searching the most discriminatory loci that define a particular phylogenetic tree. We could not detect any particular genomic trait related with the four LasR deficient virulent *P. aeruginosa* isolates, showing that strains with this modified QS response can be selected from any strain belonging to Clades 1 and 2 and do not represent an homogeneous genomic group. In addition, we identified ten highly discriminatory loci that recreate the wgMLST phylogeny and show that two of these loci encode for proteins that are global regulators of virulence related traits, and one encodes a type III effector protein. These results show that the ability to establish pathogenic interactions is an essential part of *P. aeruginosa* life-style that has played an important part in the evolution of this bacterial species.

## METHODS

### Determination of *P. aeruginosa* virulence in a mouse model

The assay to measure virulence of *P. aeruginosa* INP43 strain in the mouse model was done as described previously [9].

### Identification of mutations affecting LasR activity

The *lasR, lasI, vfr and cyaB* coding sequences were retrieved, translated into proteins and used as queries in BLASTP version 2.2.26+alignment with the correspondent PAO1 sequence [19]. The sequence of regulatory sequence was analysed using blast analysis.

### Analysis of genomes using cano-wgMLST_BacCompare software

**Fig. 1.**
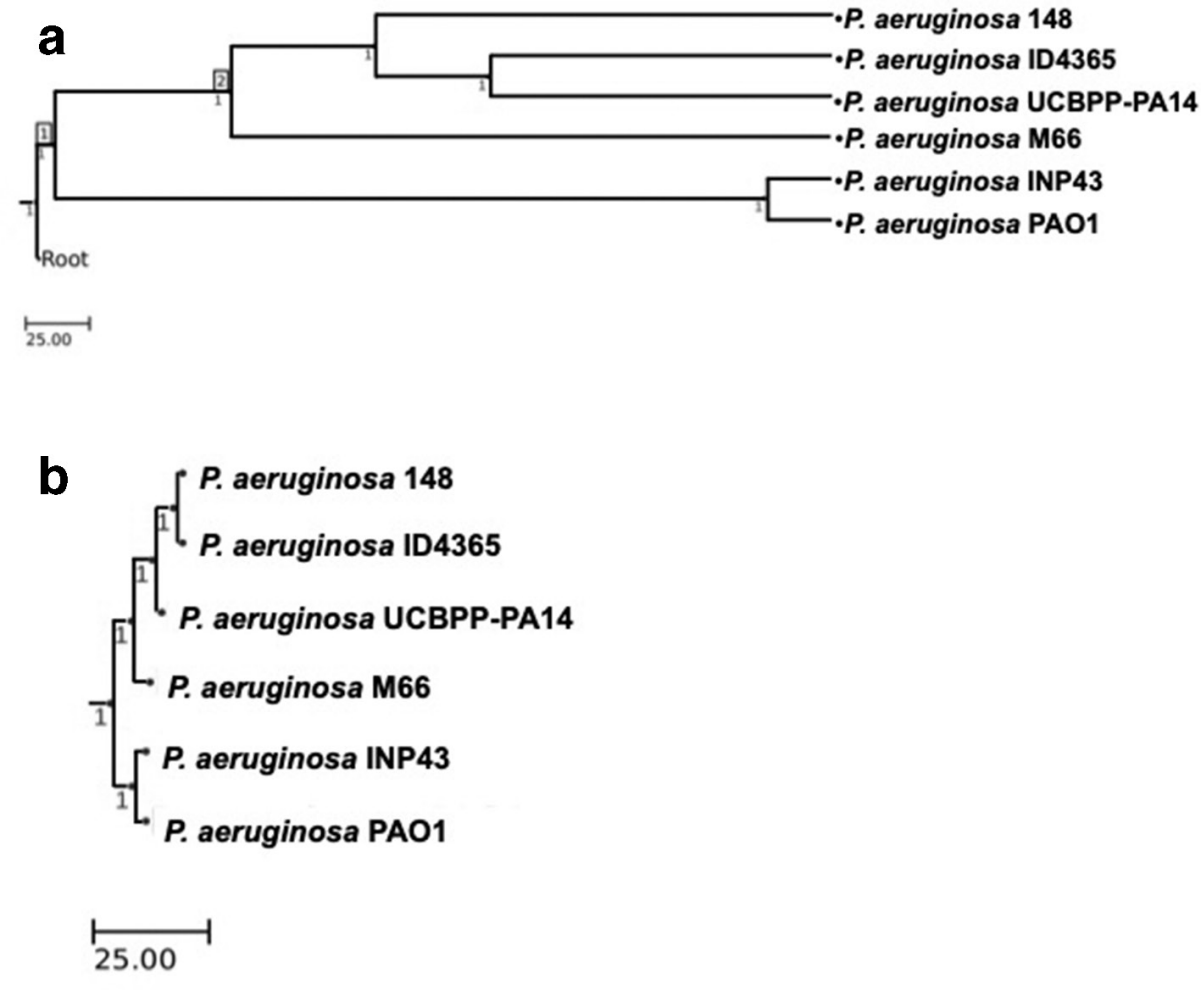
Phylogenetic analysis of strains studied in this work. (a) Phylogenetic tree built based on ws(MLST); (b) phylogenetic tree built based on the homology of the ten highly discriminatory loci identified in this work.

**Fig. 2.**
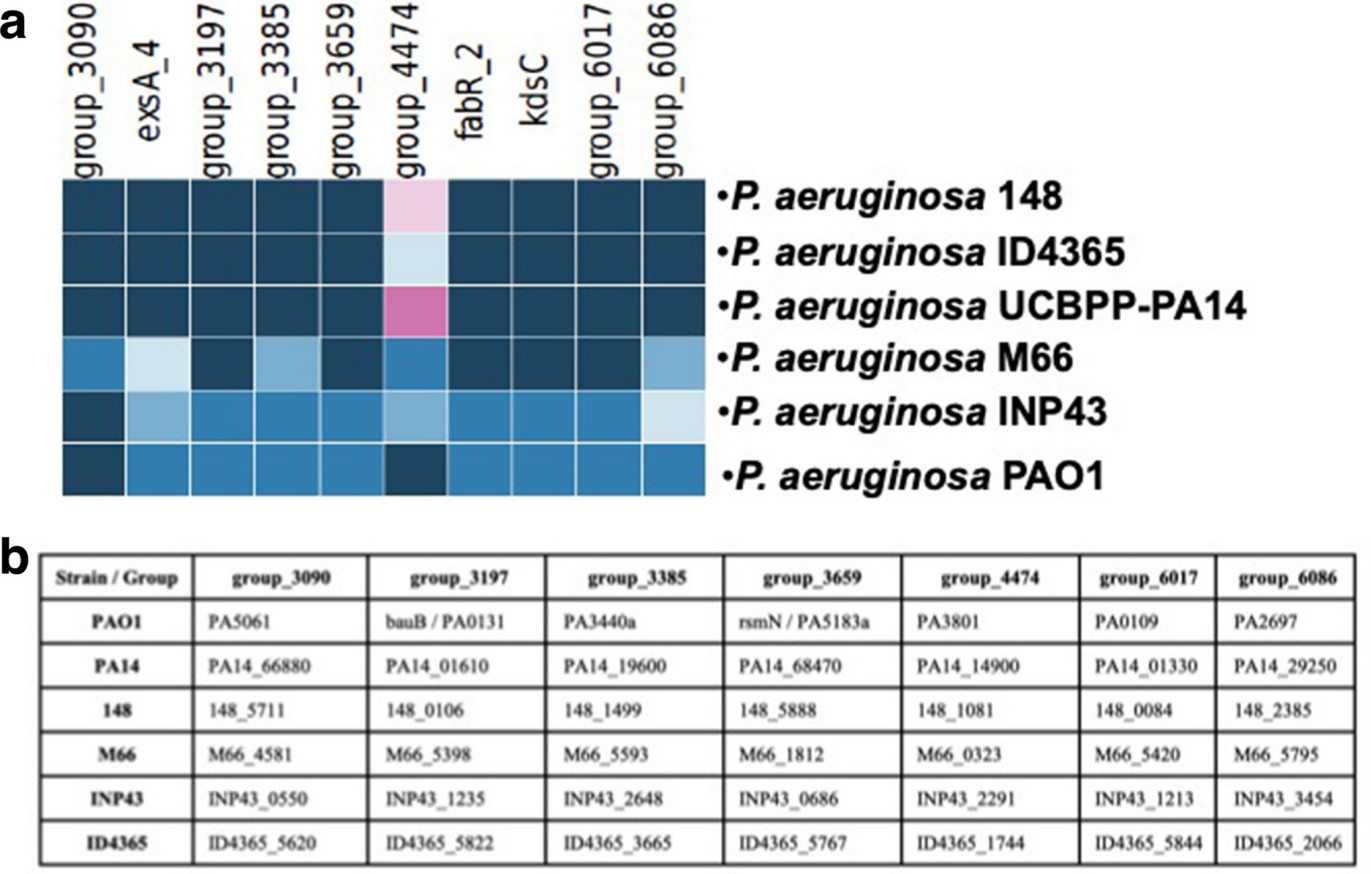
Description of the ten highly discriminatory loci identified in this work. (a) Heatmap of the ten highly discriminatory loci identified in this work. The same color shows that the locus is conserved. (b) Locus number in each strain that corresponds to each genomic group shown on a.

To make the whole genome MLST analysis we used the sequences of the *P. aeruginosa* strains to be analysed (PAO1, PA14, 148, ID4365, INP43 and M66) in FASTA format. These whole genome sequences were compared through whole-genome multilocus sequence typing (wgMLST) using the cano-wgMLST_BacCompare software that was reported recently [18] to make epidemiological reaserch and comparative genomics of bacteria (available at http://baccompare.imst.nsysu.edu.tw). This software enabled us to obtain ten highly discriminatory loci that permit the distinction of strains that belong to the same species and are closely related (Figs 1 and 2, Table 2).

**Table 2.** The ten loci that are highly discriminatory between the six *P. aeruginosa* analysed strains

Gene	PAO1 ORF	Function/annotation
Group_4474	PA3801	Conserved hypothetical protein *yfgM*.
Group_3090	PA5060	Polyhydroxyalkanoate granule associated protein (phasin *phaF*)
Group_3197	PA0131	Cupin domain protein, *bauB* (beta-alanine utilization).
Group_3385	PA3440.1	HopJ type III effector protein (encodes for an uncharacterized protein in PAO1 strain).
*rsmN*	PA5183.1	RNA binding protein regulator, *rsmN*.
Group_6017	PA0109	Hypothetical protein conserved in *P. aeruginosa* and other species of *Pseudomonas *.
Group_6086	PA2697	Hypothetical protein conserved in *P. aeruginosa* and other species of *Pseudomonas *.
exsA	PA1713	Type III secretion system master regulator ExsA.
*fabR_2*	PA4890 (*desT*)	HTH-type transcriptional repressor of aerobic unsaturated fatty acids synthesis.
*kdsC*	PA4458	3-deoxy-d-manno-octulosonate 8-phosphate phosphatase KdsC (encodes for an uncharacterized protein in PAO1 strain).

### NCBI accession numbers of genomes used in this work

The accession numbers of the whole genome sequences used in the work are: NZ_SDVP00000000 (strain M66), ATAI00000000 (strain ID4365), ATAJ00000000 (strain 148), CP047592 (strain INP 43),

NC_002516 (strain PAO1) and NC_008463 (strain UCBPP-PA14).

## RESULTS AND DISCUSSION

### Characterization of the LasR defective virulent *P. aeruginosa* strains studied in this work

It has been already reported that three of the strains having an atypical QS response that were studied in this work, 148 [9, 13], ID4365 [9], and M66 [11], have an inactive LasR, but produce the RhlR/C4-HSL dependent virulence factors PYO and RL and are virulent. Here we present some further analysis of the genomic sequence of these strains (Table 1). In addition we report the characterization of strain INP43 in terms of its production of PYO and RL and its virulence in a mice model, and the mutation that causes the inactivation of its LasR (Table 1). The only one of the studied strains that does not have a mutation in *lasR* is strain M66, but it has been reported that it does not produce 3O-C12-HSL [11], the product of LasI enzyme. The transcription of *lasI* is strictly dependent on LasR activity, so this phenotype is a direct measurement of this transcriptional factor activity. The analysis of the amino acid sequence of M66 LasR, LasI, and Vfr (the CRP homolog that activates *lasR* transcription [20]), showed that they have the same sequence as the PAO1 corresponding protein. In addition the regulatory sequences of *lasR, lasI, vfr* and *cyaB* are also well conserved. However CyaB, the adenylate cyclase that produces cAMP that interacts with Vfr contains the mutation A80V compared to the PAO1 CyaB (Table 1). It is thus possible that *lasR* is not efficiently transcribed in strain M66 due to a lower cAMP level available to bind Vfr.

From these data it is clear that the four studied *P. aeruginosa* strains are LasR defective, but are able to produce PYO and RL that are dependent on RhlR/C4-HSL transcriptional activity, and are virulent in the *Galleria mellonella* or mouse models (Table 1). Therefore we conclude that the global analysis of the genomes of these strains and the PAO1 and PA14 type strains constitute a suitable model to determine whether LasR defective strains that are able to express QS-regulated traits share a particular molecular response which could be a product of a common selective pressure, as has been previously suggested [15].

### Definition of core and pangenomes of the six studied strains

The comparative analysis of the genome sequences of the six studied strains render a pangenome of 6874 loci, among which 4869 (70.8 %) constituted the core-genome, 825 loci (12 %) are present in two to five genomes and constitute the accessory genome, and 1180 (17.2 %) are only present in one genome, and are thus unique. The core genome of 17 *P *. *aeruginosa* reference strains has been reported to consist of 5233 orthologs [8], which is a slightly higher number to what we found analysing the six genomes reported here.

### Construction of a wgMLST phylogenetic tree

The core genome loci were used to construct a phylogenetic tree based on an MLST analysis (Fig. 1a). This phylogenetic tree shows that one strain studied in this work (INP43) belongs to Clade 1 and is clustered with PAO1 type strain, while the other three strains with an atypical QS-response (ID4365, 148 and M66) belong to Clade 2 and are thus clustered with PA14 type strain. It is worth noting that strain M66, which belongs to clonal group 2A isolated in 2011 at the Churince water system of Cuatro Ciénegas Coahuila, México, forms a distinct branch in the wgMLST phylogenetic tree in accordance to its position in the phylogenetic tree reported recently [11] that was constructed using FastTree 2.0 that is based on approximate maximum-likelihood [21]. These results show that the LasR deficient and virulent strains that present an atypical QS response, do not represent a particular clonal group and that this type of isolates can be part of Clade 1 or Clade 2. Thus it can be concluded that there is not a single genomic ‘pathway’ for the LasR deficient QS atypical strains, and that this type of strains are not part of an epidemiological group.

### Identification of the highly discriminatory genes that recreate the wgMLST phylogenetic tree

In order to determine what were the genes that could discriminate between the four strains with atypical QS sensing response, and the two type strains that were studied in this work, we used the cano-wgMLST_BacCompare software [18]. To do this we search for the genes that are highly discriminatory among the genes conserved in the six strains and we found ten genes (Fig. 2, Table 2) that can recreate the phylogenetic tree constructed by wgMLST (Fig. 1b). These loci (Fig. 2) include four annotated genes (*exsA, rsmN, fabR_2* and *kdsC*) and six groups defined by the cano-wgMLST_BacCompare software (group_4474, group_3090, group_3197, group_3385, group_6017, group_6086). Table 2 shows the number of the PAO1 ORF corresponding to each of the highly discriminatory loci and their characteristics, and Fig. 2b shows the number of the ORF corresponding to each group in the six genomes analysed.

The ten highly discriminatory genes that were found, present between two and six different alleles in the six strains that we studied (each allele is represented by a different colour in Fig. 2a). This reduced amount of genetic information, only ten loci, can be used as a marker of their phylogeny, since it reflects the genetic variation that explains their evolutionary pathway. Even though these highly discriminatory loci do not necessarily have a role in the evolution of these strains, it can be concluded that they in some way represent genomic markers of their phylogeny.

The presence among the ten highly discriminatory loci of two master regulators involved in the expression of genes involved in the virulence of *P. aeruginosa* (*exsA* and *rsmN*) highlights that pathogenic interactions are a fundamental part of this bacterial species way of life and not accessory traits as happens with other bacterial species.

ExsA is a master regulator involved in the expression of the type III secretion system [22], which plays an important role in *P. aeruginosa* pathogenicity [6, 7]. It is significant in this respect that group_3385 encodes for HopJ, a type III effector protein.

RsmN is an RNA binding protein that is homologous to RsmA/CsrA, but is unique to *P. aeruginosa* [23]. This family of proteins has an important role in the posttranscriptional regulation of gene expression, by binding mRNAs at a sequence near the ribosome-binding site and usually blocks their translation. The Rsm system coordinately regulates the expression of the type III secretion system [24] and of virulence factors that are regulated by quorum-sensing [25].

The ten highly discriminatory genes of the phylogeny of the six studied strains include *phaF* (group_3090, Fig. 2, Table 2), which encodes a protein that is bound to the membrane of polyhydroxyalkanoate (PHA) granules [26]. This polymer serves as a reservoir of carbon and its production is characteristic of Pseudomonads and other bacteria, so even though PHA production is not a central metabolic pathway it is a conserved trait and it is not surprising that the genetic variation of the genes involved in its production reflect *P. aeruginosa* evolution.

In addition, the ten highly discriminatory loci identified contained the *fabR_2* gene (called *desT* in PAO1, Table 2). This gene encodes a repressor of genes involved in the aerobic unsaturated fatty synthesis [27]. In addition, the ten highly discriminatory loci include two genes involved in metabolic pathways (group_3197 which is annotated as *bauB* in PAO1 and codes for an enzyme that participates in beta-alanine, and *kdsC* that encodes a 3-deoxy-d-manno-octulosonate 8-phosphate phosphatase). It is expected that genes involved in metabolic pathways, both regulatory and structural, reflect the evolutionary pathway of bacteria, so it is expected to identify this type of genes.

The three remaining loci among the ten highly discriminatory loci identified encode for hypothetical proteins, one of them (group_4474, *yfgM*) is highly conserved among a wide range of bacteria, while the other two (group_6017 and group_6086) are conserved among different *Pseudomonas* species including *P. aeruginosa *.

The high genome conservation of different *P. aeruginosa* isolates is reflected in the strict conservation of their essential genes [28]. However, this high genomic conservation is in contrast with the high phenotypic variability of different isolates [9]. The ten highly discriminatory genes that were identified in this work are not essential genes in any of the conditions that have been tested [29]. It is expected that these highly discriminatory genes are non-essential genes since they show a genetic variability that reflects the genomic variability and the evolutionary pathway of the six studied *P. aeruginosa* strains, and essential genes are expected to be less variable than the rest of the genome due to the high selective pressure that they are submitted to, and to be present in all related species independently of their lifestyle [30, 31].

## CONCLUSIONS

The *P. aeruginosa* genome is very well conserved among different isolates both of clinical and environmental origin, and the genes encoding virulence associated traits form part of its core-genome [8, 9].

Here we analyse whether four independent *P. aeruginosa* isolates that have a defective LasR, the transcriptional regulator that is the head of the QS response, have a particular genomic constitution using the cano-wgMLST_BacCompare software [18] and show that these *P. aeruginosa* isolates with an atypical QS response do not share a particular genomic profile. These results suggest that the LasR defective strains that are still able to establish pathogenic interactions can be selected from strains belonging to Clades 1 and 2 (we did not test any strain belonging to Clade 3).

The cano-wgMLST_BacCompare software allowed us to identify ten loci that are highly discriminatory and reflect the phylogeny constructed with the wgMLST of the six studied strains. Two of the identified loci (*exsA* and *rsmN*) are master regulators that are involved in the expression of the type III secretion system, and *rsmN* also in the expression of virulence traits regulated by QS. A third locus (group_3385, Tables 1 and 2) is a type III effector protein. These results show that the establishment of pathogenic interactions, and in particular the activity of the type III secretion system, is a fundamental characteristic of *P. aeruginosa* that has taken part in the evolution of this bacetrial species.
